# 

**Published:** 1864-07

**Authors:** 


### BOOKS RECEIVED.

*A Hand-Book of Uterine Therapeutics. By* EDWARD JOHN TILT, M. D., *Member of the Royal College of Physicians; Consulting Physician to the Farringdon General Dispensary; Fellow of the Royal Medical and Chirurgical Society, and of several British and Foreign Societies. New York:* WM. WOOD & Co., 61 *Walker street.*

*Transactions of the State Medical Society, of Indiana, at the Fourteenth Annual Session, held in the city of Indianapolis, May* 17 *&* 18, 1864.

*Atlantic Monthly, July Number.*—This is the first number of vol. xiv, and is a good index of what is constantly furnished the readers of this journal. A long list of contributors are announced, whose reputations are sufficient guarantee that the character and worth of the Atlantic Monthly is to be fully maintained.

*Peterson's Magazine.*—The publishers supply us regularly with this beautifully illustrated and highly entertaining magazine. Whoever is looking for the latest fashions and the most readable story should send for "Peterson."

*Godey's Lady's Book..*—This fashionable magazine is occasionally placed upon our table. We suppose the irregularity is due to the fact that we do not always acknowledge the favor, or give our readers the table of contents. We always receive thankfully such courtesies, but we do not always have time and space to speak of it.

